# Epigenome-wide association study for lifetime estrogen exposure identifies an epigenetic signature associated with breast cancer risk

**DOI:** 10.1186/s13148-019-0664-7

**Published:** 2019-04-30

**Authors:** Annelie Johansson, Domenico Palli, Giovanna Masala, Sara Grioni, Claudia Agnoli, Rosario Tumino, Maria Concetta Giurdanella, Francesca Fasanelli, Carlotta Sacerdote, Salvatore Panico, Amalia Mattiello, Silvia Polidoro, Michael E. Jones, Minouk J. Schoemaker, Nick Orr, Katarzyna Tomczyk, Nichola Johnson, Olivia Fletcher, Vittorio Perduca, Laura Baglietto, Pierre-Antoine Dugué, Melissa C. Southey, Graham G. Giles, Dallas R. English, Roger L. Milne, Gianluca Severi, Srikant Ambatipudi, Cyrille Cuenin, Veronique Chajès, Isabelle Romieu, Zdenko Herceg, Anthony J. Swerdlow, Paolo Vineis, James M. Flanagan

**Affiliations:** 1 Division of Cancer, Department of Surgery and Cancer, Faculty of Medicine, Imperial College London, 4th Floor IRDB, Hammersmith Campus, Du Cane Road, London, W12 0NN UK; 2 Cancer Risk Factors and Lifestyle Epidemiology Unit, Institute for Cancer Research Prevention and Clinical Network—ISPRO, Florence, Italy; 3 Epidemiology and Prevention Unit, Fondazione IRCCS Istituto Nazionale dei Tumori, Milan, Italy; 4 Ragusa Cancer Registry, ASP, Ragusa, Italy; 5 Unit of Cancer Epidemiology, Città della Salute e della Scienza University-Hospital and Center for Cancer Prevention (CPO), Turin, Italy; 6 Dipartimento di Medicina Clinica e Chirurgia, University of Naples Frederico II, Naples, Italy; 7 Italian Institute for Genomic Medicine, Turin, Italy; 8 The Institute of Cancer Research, London, UK; 9 Centre for Cancer Research and Cell Biology, Queen’s University Belfast, Belfast, UK; 10 The Breast Cancer Now Toby Robins Research Centre, The Institute of Cancer Research, London, UK; 11 MAP5 - UMR CNRS 8145, Université Paris Descartes, Paris, France; 12 Department of Clinical and Experimental Medicine, University of Pisa, Pisa, Italy; 13 Cancer Epidemiology and Intelligence Division, Cancer Council Victoria, Melbourne, Australia; 14 Centre for Epidemiology and Biostatistics, Melbourne School of Population and Global Health, The University of Melbourne, Melbourne, Australia; 15 Precision Medicine, School of Clinical Sciences at Monash Health, Monash University, Melbourne, Australia; 16 Genetic Epidemiology Laboratory, Department of Pathology, The University of Melbourne, Parkville, Australia; 17 Centre de Recherche en Épidémiologie et Santé des Populations (CESP, Inserm U1018), Université Paris-Saclay, UPS, UVSQ, Gustave Roussy, Villejuif, France; 18 International Agency for Research on Cancer (IARC), Lyon, France; 19 AMCHSS, Sree Chitra Tirunal Institute for Medical Sciences and Technology, Trivandrum, Kerala 695011 India; 20 Division of Breast Cancer Research, The Institute of Cancer Research, London, UK; 21 MRC-PHE Centre for Environment and Health, School of Public Health, Imperial College London, London, UK

**Keywords:** DNA methylation, EWAS, Epigenetics, Breast cancer, Cancer risk, Estrogen exposure, Hormonal exposures, Biomarker

## Abstract

**Background:**

It is well established that estrogens and other hormonal factors influence breast cancer susceptibility. We hypothesized that a woman’s total lifetime estrogen exposure accumulates changes in DNA methylation, detectable in the blood, which could be used in risk assessment for breast cancer.

**Methods:**

An estimated lifetime estrogen exposure (ELEE) model was defined using epidemiological data from EPIC-Italy (*n* = 31,864). An epigenome-wide association study (EWAS) of ELEE was performed using existing Illumina HumanMethylation450K Beadchip (HM450K) methylation data obtained from EPIC-Italy blood DNA samples (*n* = 216). A methylation index (MI) of ELEE based on 31 CpG sites was developed using HM450K data from EPIC-Italy and the Generations Study and evaluated for association with breast cancer risk in an independent dataset from the Generations Study (*n* = 440 incident breast cancer cases matched to 440 healthy controls) using targeted bisulfite sequencing. Lastly, a meta-analysis was conducted including three additional cohorts, consisting of 1187 case-control pairs.

**Results:**

We observed an estimated 5% increase in breast cancer risk per 1-year longer ELEE (OR = 1.05, 95% CI 1.04–1.07, *P* = 3 × 10^−12^) in EPIC-Italy. The EWAS identified 694 CpG sites associated with ELEE (FDR *Q* < 0.05). We report a DNA methylation index (MI) associated with breast cancer risk that is validated in the Generations Study targeted bisulfite sequencing data (OR_Q4_vs_Q1_ = 1.77, 95% CI 1.07–2.93, *P* = 0.027) and in the meta-analysis (OR_Q4_vs_Q1_ = 1.43, 95% CI 1.05–2.00, *P* = 0.024); however, the correlation between the MI and ELEE was not validated across study cohorts.

**Conclusion:**

We have identified a blood DNA methylation signature associated with breast cancer risk in this study. Further investigation is required to confirm the interaction between estrogen exposure and DNA methylation in the blood.

**Electronic supplementary material:**

The online version of this article (10.1186/s13148-019-0664-7) contains supplementary material, which is available to authorized users.

## Background

Breast cancer is the most common women’s cancer, with an estimated 1.67 million cases diagnosed globally in 2012 [1]. Its crude incidence is rising due to an aging population and population-level changes in reproductive and lifestyle factors that affect breast cancer risk [2]. Up to 23% of breast cancer cases are considered preventable by lifestyle changes, such as maintaining a healthy weight and reducing alcohol consumption [3]. There is a need for improved risk assessment methods to target prevention and early detection to women at increased risk.

It is well established that estrogens play a role in breast cancer etiology, and women with higher circulating estrogen concentrations have an increased risk of breast cancer [4–6]. Several hormonal breast cancer risk factors contribute to a woman’s lifetime estrogen exposure. These include a younger age at menarche and an older age at menopause, which together define the reproductive span during which a woman is exposed to high levels of endogenous estrogens produced by the ovaries [7, 8]. The number of pregnancies is associated with a long-term decrease in both estrogen exposures and breast cancer risk [9, 10]. A small decrease in risk is seen for women who breastfeed for longer, and this decreases the total lifetime estrogen exposure [11, 12]. Exogenous hormones provided by oral contraceptives (OC) and hormone replacement therapy (HRT) increase risk during use, but risk returns to that for unexposed women 5–10 years after cessation [13–17]. Additionally, lifestyle risk factors for breast cancer such as higher postmenopausal body mass index (BMI), alcohol consumption, physical inactivity, and smoking can each affect circulating estrogen concentrations [4, 18–22].

Epigenetic mechanisms such as DNA methylation control gene expression and may be influenced by environmental and lifestyle exposures. Epigenome-wide association studies (EWAS) of blood DNA methylation for breast cancer risk measured have identified associations with global hypomethylation and several candidate genes, but these have generally not been replicated across studies [23–26]. Numerous large EWAS have identified epigenetic signatures for smoking [27], alcohol consumption [28], BMI [29], and aging [30, 31], and hypomethylation signatures associated with smoking can improve the prediction of lung cancer [32, 33]. We propose that an EWAS of breast cancer risk factors may identify CpG sites that could be used in risk prediction models [23]. We hypothesize that estrogen exposures over the lifetime give rise to accumulated changes in DNA methylation, detectable in the blood, which might add useful information to breast cancer risk prediction.

The aims of this study were to identify a DNA methylation signature reflecting a woman’s lifetime estrogen exposure and assess the signatures’ association with breast cancer risk. There is no standard model to estimate a woman’s total lifetime estrogen exposure, and numerous approaches have been used [34–39]. For example, the Pike model reflecting the “breast tissue aging” in relation to breast cancer risk includes a woman’s age at menarche, age at first full-term pregnancy, and age at menopause modeling changes over time [38]. In this study, we have used an estimated lifetime estrogen exposure (ELEE) model that reflects the reproductive span, comprising a woman’s time between age at menarche and age at menopause minus 1 year for each pregnancy and duration of breastfeeding, calculated at the time at recruitment. We performed an EWAS and identified 694 CpG sites associated with ELEE. We then developed a methylation signature of ELEE that showed association with breast cancer risk and was further validated in a large independent study cohort using targeted bisulfite sequencing, and a meta-analysis of three additional independent study cohorts.

## Methods

### Study cohorts

Data from two independent prospective cohort studies were used for the primary analysis: the Italian cohort from the European Prospective Investigation into Cancer and Nutrition study (EPIC-Italy) and the UK-based Generations Study. Questionnaire data and blood samples were collected at the time of study enrollment. EPIC-Italy included epidemiological questionnaire data from 32,059 women (dataset 1) and peripheral blood DNA methylation data measured using the HM450K array for 162 matched pairs of incident breast cancer cases and controls (dataset 2) [24]. The Generations Study cohort included HM450K peripheral blood DNA array data for a subset of 92 healthy women (dataset 3) [40] and 440 matched pairs of incident breast cancer cases and controls (independent from the HM450K dataset), who provided blood DNA samples used for targeted bisulfite sequencing (dataset 4). Inclusion criteria for incident breast cancer cases in the Generations Study, with blood samples taken prior to diagnosis, were the following: invasive ER-positive breast cancer with no previous history of (non-breast) cancer, white ethnicity, and completeness of epidemiological data. Controls were individually matched to cases on age at blood draw ± 5 years. Additional replication cohorts used in the meta-analysis included an additional 118 case-control pairs from EPIC-Italy (dataset 5), 435 case-control pairs from EPIC-IARC (dataset 6) [41], and 310 case-control pairs from the MCCS (dataset 7) [42]. Further information for these study cohorts is provided in Additional file 1: Supplementary material and methods.

### EWAS of ELEE

Different ELEE models including a woman’s reproductive span (age at menopause minus age at menarche for postmenopausal women and age at recruitment minus age at menarche for premenopausal women), number of pregnancies, and breastfeeding duration were considered. The models were assessed for association with breast cancer risk in EPIC-Italy (*n* = 1193 cases and 30,671 controls, Additional file 2: Table S1) using age-adjusted Cox regression. The ELEE model selected for the EWAS included as many of these risk factors as possible without reducing the significance of the association with breast cancer for pre- and postmenopausal women. An EWAS with ELEE as the exposure and DNA methylation as the outcome was conducted for EPIC-Italy using a beta regression model on HM450K beta values. Subjects with missing information for ELEE were excluded (*n* = 87), as were cases with age at diagnosis < 50 (*n* = 28), to enrich for ER-positive disease, leaving 216 women for the EWAS (Additional file 2: Table S2). Potential confounders, known to influence methylation or estrogen levels, were adjusted for; these included age, BMI, alcohol consumption, and smoking duration, all reported at recruitment, as well as technical confounders including batch, position on batch, and white blood cell (WBC) composition [43]. Multiple testing was accounted for using the false discovery rate (FDR) *Q* values in R function “p.adjust.” Beta regression coefficients are not interpretable as methylation percentage changes. Therefore, to get interpretable estimates for the significantly associated CpG sites, i.e., percentage change in DNA methylation per unit longer ELEE, a linear mixed-effects regression model adjusted for the same variables, including random effects for batch and position on chip, was applied to beta values multiplied by 100.

### Laboratory analysis

HM450K array data generation has been described previously [24, 40–42]. For validation, targeted bisulfite sequencing was conducted in the Generations Study (*n* = 880) using the Fluidigm 48.48 Access Array. Forty-two CpG sites for validation were selected from the EWAS based on the magnitude of change in DNA methylation (> 0.1%) per 1-year longer ELEE (mixed-effects linear regression model coefficient) and statistical significance of association with ELEE (beta regression model *P* value < 7 × 10^−5^). To estimate WBC composition, five HM450K CpG probes that showed independent correlation with five different WBC types were included in the target panel (Additional file 3: Figure S1). The 880 Generations Study samples were sequenced on the Illumina MiSeq in 20 batches in four sequencing pools (6, 5, 5, and 4 batches). After quality control, two batches were rerun (batches 12 and 19) due to poor sequencing data, likely a result of decreased performance for the barcodes used for these batches. In the results, only the sequencing data from the new batches 12 and 19 are used, referring to sequencing pool 5. DNA methylation levels were extracted using Bismark [44] and analyzed in R version 3.3.2.

### Statistical analysis

Quality control (QC) of the targeted sequencing data was conducted to exclude CpG sites (*n* = 42) with low coverage or with large difference in DNA methylation levels between duplicated pairs (*n* = 31 CpG sites passing quality control). A methylation index (MI) of ELEE was developed using the same 31 CpG sites in the HM450K data with complete information for ELEE from both EPIC-Italy (dataset 2, *n* = 237) and the Generations Study (dataset 3, *n* = 65, Additional file 2: Table S2). To develop the MI, ridge regression was conducted in a 10-fold cross-validation repeated 100 times, using the R package “glmnet” in “train” in the R package “caret” to estimate the following parameters: penalty coefficient (lambda) for ridge regression and regression coefficients for the model. The final MI model for predicted ELEE was calculated as a linear function, i.e., intercept plus the sum of the DNA methylation levels at the CpG sites included in the model weighted by their coefficient. The correlation between the MI and ELEE was evaluated in the Generations Study targeted sequencing data (dataset 4, pairs with coverage > 30 sequence reads for at least 10% of the remaining CpG sites) using the Pearson’s correlation coefficient. The association between the MI and risk of breast cancer was assessed using conditional logistic regression (R function “clogit” in the package “survival”) for matched case-control pairs in EPIC-Italy HM450K development data (dataset 2, *n* = 162 pairs), the Generations Study targeted sequencing validation data (dataset 4), and in each of the additional cohorts for the meta-analysis including EPIC-Italy (dataset 5, *n* = 118 pairs), EPIC-IARC (dataset 6, *n* = 420 pairs), and MCCS (dataset 7, *n* = 310 pairs). The meta-analysis of log odds ratios and standard errors was conducted using a weighted random-effects model, applying the restricted-maximum likelihood method (function “rma.uni” in the R package “metafor”). The Cochran’s *Q* statistics and *I*^2^ statistics were used to estimate heterogeneity between the studies; *Q* < 0.05 and *I*^2^ > 50% were defined as heterogeneous estimations. If the heterogeneity *I*^2^ was equal to 0, the meta-analysis behaved as a fixed-effects model. The ORs for all models were adjusted for baseline age, BMI, alcohol consumption, smoking duration, and estimated WBC composition in a multivariable model. Additionally, for each of the target CpG sites passing QC, the association with breast cancer risk was investigated using conditional logistic regression. Further details on methods and workflow are described in Additional file 1: Supplementary material and methods and Additional file 3: Figure S2.

## Results

### The ELEE is associated with breast cancer risk in EPIC-Italy

The estimated lifetime estrogen exposure (ELEE) was calculated as a woman’s reproductive span (age at menopause or, for premenopausal women, age at recruitment, minus age at menarche) minus 1 year per pregnancy and breastfeeding duration before recruitment in years. For EPIC-Italy (dataset 1, *n* = 1193 cases and 30,671 controls, Additional file 2: Table S1), a 1-year longer ELEE, ranging from 5 to 49, was associated with a 5% increase in breast cancer risk (hazard ratio (HR) = 1.05, 95% confidence interval (CI) 1.04–1.07, *P* = 3 × 10^−12^, Additional file 2: Table S3) and was associated with breast cancer risk for both pre- and postmenopausal women (HR = 1.09, 95% CI 1.03–1.15, *P* = 0.002, and HR = 1.03, 95% CI 1.01–1.05, *P* = 6 × 10^−4^, Additional file 2: Table S3).

### EWAS identifies CpG sites associated with ELEE in EPIC-Italy

**Fig. 1:**
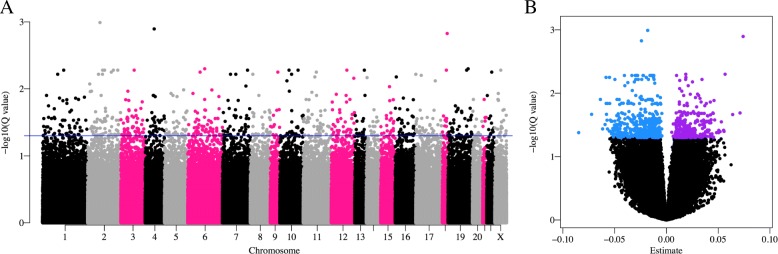
EWAS identifies CpG sites significantly associated with ELEE in EPIC-Italy. An EWAS with DNA methylation (HM450K beta values) as outcome and ELEE as exposure was conducted in EPIC-Italy (*n* = 216) using a beta regression model adjusted for age, BMI, alcohol consumption, and smoking duration (all variables reported at recruitment), and batch, position on batch, and WBC composition. **a** Manhattan plot of minus log_10_*Q* values for FDR-corrected *P* values from the EWAS of all 404,596 probes across the genome. Blue line indicates FDR *Q* value threshold of 0.05. **b** Volcano plot of the regression coefficients (estimates) from the beta regression model, showing significant hypomethylated (in blue) and hypermethylated probes (in purple)

The EWAS of ELEE conducted in EPIC-Italy (dataset 2, *n* = 216, Additional file 2: Table S2) gave *P* values that were moderately inflated (Additional file 3: Figure S3), with a genomic inflation factor lambda of 1.13. After correction for multiple testing, the EWAS identified 694 CpG probes associated with ELEE (FDR *Q* < 0.05, Fig. 1a) with a mix of hypo- and hypermethylated CpG probes (Fig. 1b). Two sensitivity analyses were conducted firstly, including all cases and controls with complete information of ELEE (*n* = 237), and secondly for controls only (*n* = 119), and estimates from each analysis were highly correlated (*r* = 0.997 and *r* = 0.963, respectively, Additional file 3: Figure S4). All CpG probes were associated with ELEE (*Q* < 0.05) in the first analysis and 563 in the second analysis with controls only. Out of the 694 CpG probes associated with ELEE, CpG sites were selected for the targeted bisulfite sequencing. The selection was based on the largest magnitude of change in DNA methylation (> 0.1%) and statistical significance (*P* < 7 × 10^−5^). Furthermore, poorly performing assays were excluded before the sequencing in the Generations Study. A total of 42 CpG sites were included in the final target panel for targeted bisulfite sequencing in the Generations Study using the Fluidigm 48.48 Access Array (Table 1).

**Table 1:** List of the 42 target CpG sites included in the targeted bisulfite sequencing

HM450K probe	Chr	Position	Nearest gene	Distance to gene	EWAS of ELEE in EPIC-Italy^b^			
					Estimate	SE	*P* value	*Q* value
cg01893629	chr12	34494825	ALG10	313588	− 0.14	0.03	8.15E−05	4.96E−02
cg08254089^a^	chr20	36933189	BPI	0	− 0.28	0.08	7.04E−05	4.78E−02
cg21590238	chr12	121454837	C12orf43	536	− 0.29	0.08	2.67E−06	1.44E−02
cg21153102	chr15	41252147	CHAC1	3429	− 0.43	0.14	6.16E−05	4.58E−02
cg03340215^a^	chr15	83315615	CPEB1	0	− 0.31	0.08	4.78E−05	4.21E−02
cg06968859	chr2	80724209	CTNNA2	0	− 0.26	0.05	2.52E−09	1.02E−03
cg12105860^a^	chr12	31742801	DENND5B	0	0.14	0.03	1.58E−06	1.21E−02
cg16840364^a^	chr4	84539569	GPAT3	12541	− 0.14	0.06	4.77E−05	4.21E−02
cg08835688	chr7	50849931	GRB10	0	− 0.14	0.03	4.50E−05	4.11E−02
cg08349826	chr16	10346403	GRIN2A	69791	− 0.11	0.04	4.19E−05	4.02E−02
cg23681866	chr6	29895175	HLA-J	0	− 1.33	0.31	2.17E−05	3.13E−02
cg22968966	chr16	22959875	HS3ST2	32215	− 0.40	0.12	7.95E−05	4.92E−02
cg15127563	chr2	231729487	ITM2C	132	0.36	0.09	1.77E−05	2.95E−02
cg20020161	chr2	231732669	ITM2C	0	− 0.14	0.04	4.49E−05	4.11E−02
cg22097768	chr17	61615913	KCNH6	0	− 0.20	0.05	4.88E−05	4.25E−02
cg17969123	chr19	18745971	KLHL26	1865	− 0.15	0.04	3.18E−05	3.65E−02
cg05422360	chrX	75648455	MAGEE1	0	− 0.44	0.12	1.72E−07	5.26E−03
cg01768446	chr16	89982419	MC1R	1866	− 0.13	0.03	4.57E−05	4.13E−02
cg25372296	chr1	98510328	MIR137HG	0	0.33	0.09	3.83E−06	1.65E−02
cg04519403^a^	chr5	79298951	MTX3	11862	− 0.24	0.05	7.51E−06	2.20E−02
cg12091786	chr20	61877942	NKAIN4	0	− 0.37	0.10	2.07E−05	3.06E−02
cg25279613^a^	chr7	24956523	OSBPL3	0	0.20	0.06	4.95E−06	1.96E−02
cg24536703	chr11	77183438	PAK1	0	− 0.32	0.09	2.71E−05	3.43E−02
cg24036523	chr14	73712256	PAPLN	0	− 0.45	0.11	3.05E−05	3.65E−02
cg16720405	chr3	122790178	PDIA5	0	− 0.17	0.05	5.17E−05	4.33E−02
cg13674411	chr1	204232677	PLEKHA6	0	0.11	0.03	3.50E−05	3.76E−02
cg20684174	chr11	7541255	PPFIBP2	0	− 0.13	0.03	1.23E−05	2.67E−02
cg01430588^a^	chr17	56769767	RAD51C	194	− 0.32	0.09	6.04E−05	4.51E−02
cg22273487	chr20	32580931	RALY	525	0.19	0.05	1.60E−05	2.84E−02
cg22343083	chr8	54786401	RGS20	0	− 0.30	0.07	1.92E−06	1.26E−02
cg22758104	chr17	50465	RPH3AL	11713	− 0.23	0.06	4.62E−07	6.07E−03
cg16733643^a^	chr1	41575522	SCMH1	0	− 0.42	0.11	5.00E−05	4.25E−02
cg17588491	chr22	25198892	SGSM1	3242	− 0.17	0.04	4.41E−05	4.06E−02
cg13971030	chr11	35366721	SLC1A2	0	− 0.49	0.14	9.19E−06	2.41E−02
cg17567562^a^	chr3	47687980	SMARCC1	0	− 0.48	0.11	1.18E−05	2.66E−02
cg10298859	chr13	112883993	SPACA7	146656	− 0.20	0.04	2.26E−07	5.26E−03
cg19216791	chr19	5568216	TINCR	210	− 0.25	0.07	4.13E−05	4.02E−02
cg25936380	chr2	120981591	TMEM185B	606	− 0.29	0.07	2.22E−07	5.26E−03
cg01824466^a^	chr8	95959531	TP53INP1	0	− 0.23	0.08	8.08E−05	4.96E−02
cg26657235	chr6	150378972	ULBP3	4367	− 0.12	0.03	1.32E−05	2.76E−02
cg08551047	chr15	91473569	UNC45A	0	− 0.58	0.14	2.87E−06	1.46E−02
cg20394620^a^	chrX	48541924	WAS	260	− 0.20	0.05	3.02E−06	1.46E−02

*SE* standard error

^a^Marked probes did not pass quality control in the targeted sequencing data and were not included in the analysis of the methylation index

^b^Result from the EWAS of ELEE in EPIC-Italy (*n* = 216, dataset 2). The estimates correspond to regression coefficients from a mixed-effects linear regression model (percentage change in DNA methylation per unit longer ELEE), and *P* values from the beta regression model, which have been corrected for multiple testing using FDR (*Q* values)

### Quality control of targeted sequencing data prior to model development

Targeted bisulfite sequencing of 42 target regions using the Fluidigm 48.48 Access Array was conducted for 880 samples (440 matched case-control pairs) from the Generations Study (dataset 4). The targeted sequencing data was of high quality: average sequencing depth per CpG site per sample was 1740, > 97% of the reads were assigned to a sample (i.e., had a barcode sequence), and > 99% of the paired reads were aligned to target regions (Additional file 2: Table S4). Additionally, a high correlation in DNA methylation values was observed between batches, but with some variability in some of the assays (mean *r* = 0.88). Eleven CpG sites were excluded from the analysis due either to low coverage across batches or to high variation in DNA methylation levels between duplicated pairs. Out of the 880 samples, subjects were excluded due to incorrect case-control status (1 pair) or low coverage in > 10% remaining target CpG sites (*n* = 100 matched pairs where at least one sample of the pair had low coverage), leaving a total of 678 samples (339 matched case-control pairs, Table 2) for the analyses. In the 328 matched case-control pairs in the Generations Study with coverage > 30 for all five WBC CpG sites, no difference (*P* > 0.05) in DNA methylation levels between cases and controls were observed (Additional file 3: Figure S1).

**Table 2:** Table of characteristics for case-control pairs in EPIC-Italy and the Generations Study

		EPIC-Italy		The Generations Study		
		Cases (*n* = 162)	Controls (*n* = 162)	Cases (*n* = 339)	Controls (*n* = 339)	*P* ^b^
Age	Mean (st.dev.), years	52.9 (7.2)	53.0 (7.1)	53.9 (10.3)	54.1 (10.4)	0.054
Time to diagnosis	Mean (st.dev.), years	5.3 (4.4)	NA	4.0 (2.4)	NA	*0.022*
Menopausal status	*n* (%)					0.690
Premenopausal		52 (32.1%)	49 (30.2%)	135 (39.8%)	127 (37.5%)	
Postmenopausal		85 (52.5%)	87 (53.7%)	204 (60.2%)	212 (62.5%)	
Age at menarche	Mean (st.dev.), years	12.7 (1.4)	12.7 (1.7)	12.7 (1.4)	12.7 (1.5)	0.914
Age at menopause	Mean (st.dev.), years	50.2 (3.7)	49.1 (3.8)	50.3 (4.3)	50.1 (4.5)	0.126
Number of pregnancies	Mean (st.dev.)	1.6 (1.1)	1.7 (1.0)	1.9 (1.1)	1.9 (1.2)	*4 × 10* ^*−4*^
Ever breastfed	*n* (%)	103 (63.6%)	112 (69.1%)	265 (78.2%)	274 (80.8%)	0.179
Breastfeeding duration	Mean (st.dev.), years	0.7 (0.6)	0.8 (0.7)	0.9 (1.0)	0.8 (0.9)	*2 × 10* ^*−5*^
BMI	Mean (st.dev.), kg/m^2^	25.8 (4.1)	25.3 (4.3)	25.7 (4.3)	25.2 (4.3)	0.626
Alcohol consumption^a^	Mean (st.dev.)	5.5 (7.0)	7.4 (9.9)	15.8 (16.7)	14.6 (15.5)	*2 × 10* ^*−26*^
Smoking status	*n* (%)					*8 × 10* ^*−10*^
Smoker		31 (19.1%)	36 (22.2%)	25 (7.4%)	23 (6.8%)	
Former		23 (14.2%)	41 (25.3%)	98 (28.9%)	86 (25.4%)	
Never		106 (65.4%)	85 (52.5%)	216 (63.7%)	230 (67.9%)	
Smoking duration	Mean (st.dev.), years	8.1 (12.6)	10.6 (13.4)	4.4 (9.0)	3.9 (9.0)	*4 × 10* ^*−10*^
OC ever	*n* (%)	59 (36.4 %)	67 (41.4 %)	253 (74.6%)	256 (75.5%)	*< 2 × 10* ^*−16*^
OC duration	Mean (st.dev.), years	4.1 (5.1)	5.6 (5.8)	8.1 (6.4)	8.8 (6.8)	*4 × 10* ^*−9*^
HRT ever	*n* (%)	23 (14.2 %)	28 (17.3 %)	239 (70.5%)	241 (71.1%)	*2 × 10* ^*−7*^
HRT duration	Mean (st.dev.), years	2.8 (2.7)	2.6 (3.4)	6.7 (5.7)	5.4 (4.3)	*5 × 10* ^*−8*^

^a^Alcohol consumption in the Generations Study reported in average units per week, converted to average gram per day by multiplying with 8 (1 unit = 8 g alcohol) and dividing by 7

^b^*P* values indicate differences between all subjects from EPIC-Italy and all subjects from the Generations Study: *t* test for continuous variables, chi-squared test for categorical variables

### The MI is associated with breast cancer risk

**Fig. 2:**
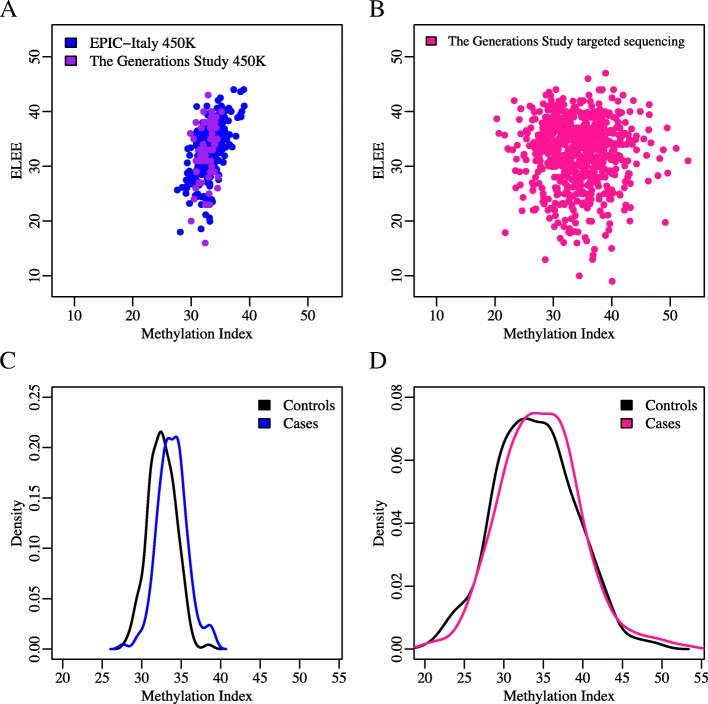
The MI is associated with breast cancer risk. The MI was developed in combined HM450K data from EPIC-Italy (dataset 2, *n* = 237) and the Generations Study (dataset 3, *n* = 65) using ridge regression. The correlation between the MI and ELEE and the association between the MI and breast cancer risk were evaluated. **a** The correlations between the MI and ELEE in the development of HM450K data were as follows: *r* = 0.60 and *P* = 6 × 10^−25^ for EPIC-Italy and *r* = 0.27 and *P* = 0.027 for the Generations Study **b** The MI and ELEE were not correlated in the Generations Study targeted sequencing data (*r* =− 0.04, *P* = 0.340). **c** Density plot of the MI values in controls and cases in EPIC-Italy HM450K data. The MI was significantly associated with breast cancer risk in EPIC-Italy (*n* = 162 pairs, OR = 1.51, 95% CI 1.26–1.82, *P* = 1 × 10^−5^). **d** Density plot of the MI values in controls and cases in the Generations Study targeted sequencing data. The MI was significantly associated with breast cancer risk in the Generations Study (*n* = 339 pairs, OR = 1.04, 95% CI 1.01–1.08, *P* = 0.022). ORs were adjusted for age, BMI, alcohol consumption, and smoking duration (all variables reported at recruitment) and WBC composition

A methylation index (MI) to predict ELEE was developed in HM450K data from EPIC-Italy (dataset 2, *n* = 237) and the Generations Study (dataset 3, *n* = 65, Additional file 2: Table S2) using ridge regression on the 31 target CpG sites passing QC in the targeted bisulfite sequencing data. As expected, the MI correlated with ELEE in the development data; a high correlation was observed in EPIC-Italy (*r* = 0.60, *P* = 6 × 10^−25^) and moderately correlated in the Generations Study HM450K data (*r* = 0.27, *P* = 0.027, Fig. 2a). The correlation between the MI and ELEE, however, was not replicated in the Generations Study targeted sequencing data (*n* = 678, *r* = − 0.04, *P* = 0.340, Fig. 2b). We also observed no association between the ELEE and breast cancer risk in the Generations Study (*n* = 339 matched case-control pairs, age-adjusted odds ratio (OR) = 1.01, 95% CI 0.98–1.04, *P* = 0.562), in contrast to EPIC-Italy subjects from the HM450K dataset (*n* = 162 matched case-control pairs, age-adjusted OR = 1.10, 95% CI 1.03–1.17, *P* = 0.007). The correlations between the MI and ELEE were similar for pre- and postmenopausal women in the Generations Study, but stronger for postmenopausal women (*r* = 0.72, *P* = 2 × 10^−22^) than for premenopausal women (*r* = 0.53, *P* = 7 × 10^−7^) in EPIC-Italy (test for heterogeneity between pre- and postmenopausal women in EPIC-Italy: *Q* = 0.79, *I*^2^ = 0 (no heterogeneity); Generations Study: *Q* = 0.42, *I*^2^ = 0 (no heterogeneity)).

The association between the MI and breast cancer risk was tested using matched case-control pairs from EPIC-Italy HM450K data and the Generations Study targeted sequencing data (*n* = 162 and 339 pairs respectively, Table 2). In a multivariable model, each unit increase in the MI, ranging from 27.6 to 39.1, was associated with a 51% increase in breast cancer risk in EPIC-Italy (OR = 1.51, 95% CI 1.28–1.82, *P* = 1 × 10^−5^, Fig. 2c). The association was validated in the Generations Study targeted sequencing data: A comparable analysis of the Generations Study data gave an estimated 4% increase in risk per unit increase in MI, which ranged from 20.3 to 53.1 (OR = 1.04, 95% CI 1.01–1.08, *P* = 0.022, Fig. 2d). The OR estimate was greater for postmenopausal women in both EPIC-Italy and the Generations Study (OR = 1.91, 95% CI 1.29–2.82, *P* = 0.001 and OR = 1.07, 95% CI 1.02–1.12, *P* = 0.006, respectively) than for premenopausal women (OR = 1.61, 95% CI 1.17–2.22, *P* = 0.004 and OR = 1.01, 95% CI 0.94–1.09, *P* = 0.713, respectively). However, the test for heterogeneity between pre- and postmenopausal women in EPIC-Italy, *Q* = 0.19 and *I*^2^ = 43, and Generations Study, *Q* = 0.24 and *I*^2^ = 27, suggested no significant heterogeneity.

Sensitivity analysis on the model development was also conducted using controls only in the combined EPIC-Italy and the Generations Study HM450K data (datasets 2 and 3, *n* = 184). The control-only MI model showed a very similar association with breast cancer risk as previously when tested on the case-control pairs from the Generations Study (*n* = 339 pairs, OR = 1.04 per unit increase in MI, 95% CI 1.01–1.08, *P* = 0.012).

### Meta-analysis of the association between MI and breast cancer risk

**Fig. 3:**
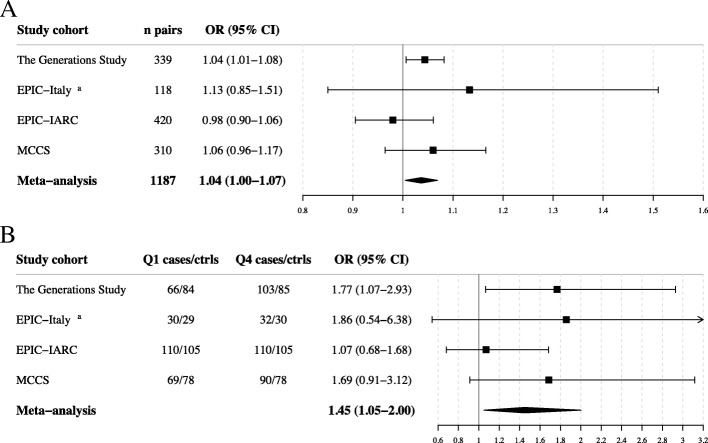
Meta-analysis of the association between MI and breast cancer risk. The association between MI and risk for breast cancer, as a continuous variable (**a**) or as a categorical variable (**b**), was estimated in the four studies included in the meta-analysis using conditional logistic regression adjusted for age, BMI, alcohol consumption, and smoking duration (all variables reported at recruitment) and WBC composition. The log odds ratios were combined in a meta-analysis using restricted-maximum likelihood model. The square boxes represent the odds ratios (ORs) and the lines the 95% confidence intervals (CIs). ^a^EPIC-Italy corresponds to the new EPIC-Italy samples, not included in the development of the MI

The association between the MI and breast cancer risk was examined in a meta-analysis, excluding the discovery data from EPIC-Italy. The analysis included 2374 women (1187 matched case-control pairs) from four prospective study cohorts, the Generations Study targeted sequencing data, additional subjects from EPIC-Italy, EPIC-IARC, and MCCS, with mean time to diagnosis of 4.0, 8.5, 7.5, and 7.9 years, respectively (Additional file 2: Table S7). All estimates were adjusted for baseline age, BMI, smoking duration, alcohol consumption, and WBC composition. The combined meta-analysis for MI as a continuous variable showed low heterogeneity across study cohorts (*Q* = 0.45, *I*^2^ = 0%) and an association with breast cancer risk, with 4% increase in risk per one unit increase in the MI (OR = 1.04, 95% CI 1.00–1.07, *P* = 0.024, Fig. 3a). Women in the highest quartile compared with the lowest quartile of MI had higher breast cancer risk with a combined OR of 1.45 (OR = 1.45, 95% CI 1.05–2.00, *P* = 0.024, Fig. 3b) and low heterogeneity (*Q* = 0.44, *I*^2^ = 16%). There was no significant association between the MI and breast cancer risk between pairs with a shorter time to diagnosis (less than median) in the combined meta-analysis (OR = 1.03, 95% CI 0.98–1.08, *P* = 0.241), but there was a significant association in pairs with time to diagnosis above the median (OR = 1.05, 95% CI 1.01–1.10, *P* = 0.021) (Additional file 2: Table S8). Lastly, the MI did not correlate with the ELEE in any of these four study cohorts (Additional file 2: Table S9).

To explore non-linearity in the association between the MI and breast cancer risk, the MI was stratified into quartiles defined by the distribution in controls in each cohort. A higher breast cancer risk was observed for women in the highest quartile compared with those in the lowest quartile in EPIC-Italy (OR_Q4_vs_Q1_ = 5.45, 95% CI 2.17–13.67, *P* = 3 × 10^−4^) and in the Generations Study (OR_Q4_vs_Q1_ = 1.77, 95% CI 1.07–2.93, *P* = 0.027, Additional file 2: Table S5), but not in three additional cohorts. In the meta-analysis, excluding the EPIC-Italy development data, increased breast cancer risk was observed for women in the highest quartile compared with the lowest quartile (OR = 1.45, 95% CI 1.05–2.00, *P* = 0.024) and modest heterogeneity (*Q* = 0.44; *I*^2^ = 16%, Additional file 2: Table S5).

**Fig. 4:**
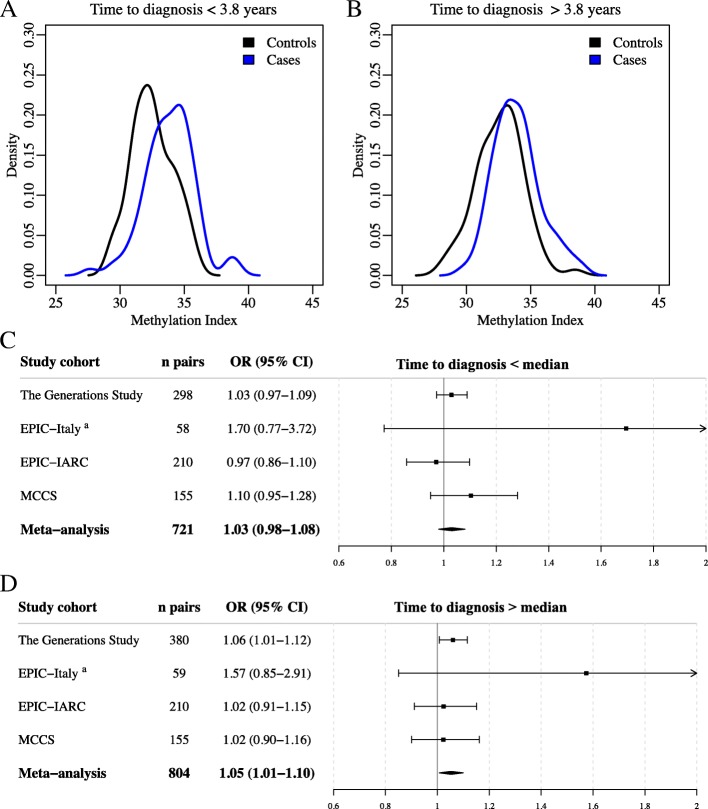
Time to diagnosis and the association between the MI and breast cancer risk. Matched case-control pairs were stratified on median time to diagnosis in EPIC-Italy HM450K data (dataset 2) and in the four study cohorts included in the meta-analysis. The association between the MI and breast cancer risk was analyzed in the two groups. **a** The MI was significantly associated with breast cancer risk in EPIC-Italy pairs with a shorter time to diagnosis (*n* = 81 pairs, OR = 1.47, 95% CI 1.12–1.93, *P* = 0.005). **b** The MI was significantly associated with breast cancer risk in EPIC-Italy pairs with a longer time to diagnosis (*n* = 81 pairs, OR = 1.84, 95% CI 1.30–2.61, *P* = 0.001). **c** The combined meta-analysis including pairs with shorter time to diagnosis showed no significant association between the MI and breast cancer risk (OR = 1.03, 95% CI 0.98–1.08, *P* = 0.241). **d** The combined meta-analysis including pairs with shorter time to diagnosis showed no significant association between the MI and breast cancer risk (OR = 1.05, 95% CI 1.01–1.10, *P* = 0.021). The log odds ratios were combined in the meta-analyses using restricted-maximum likelihood model. ORs were adjusted for age, BMI, alcohol consumption, and smoking duration (all variables reported at recruitment) and WBC composition

Reverse causation would be indicated if the association between MI and breast cancer risk was higher in cases with a short time to diagnosis. To explore this, we investigated the association between the MI and breast cancer risk stratified by median time to diagnosis in cases. In the EPIC-Italy development data, and in the meta-analysis, the association between the MI and breast cancer risk appeared to be stronger with longer time to diagnosis. In EPIC-Italy, the association between the MI and breast cancer risk was significant for both groups but with a higher OR for pairs with a longer time to diagnosis (*n* = 81 pairs in both groups, OR = 1.47, 95% CI 1.12–1.93, *P* = 0.005 vs OR = 1.84, 95% CI 1.30–2.61, *P* = 0.001; Fig. 4a, b). In the meta-analysis, for pairs with shorter time to diagnosis, there was no significant association with breast cancer risk (*n* = 721 pairs, OR = 1.03, 95% CI 0.98–1.08, *P* = 0.241, Fig. 4c); however, the MI was associated with breast cancer risk for pairs with a time to diagnosis above the median (*n* = 804 pairs, OR = 1.05, 95% CI 1.01–1.10, *P* = 0.021, Fig. 4d). Therefore, the data do not support reverse causation as a mechanism for the association with DNA methylation.

Individual associations between each of the 31 target CpG sites and breast cancer risk were further examined in the Generations Study (dataset 4). Four CpG sites located nearest the genes *CTNNA2*, *GRB10*, *RPH3AL*, and *TINCR* showed individual associations with breast cancer risk in a multivariable model (*P* < 0.05, Additional file 2: Table S6) and were also associated with breast cancer risk in matched case-control pairs in EPIC-Italy (dataset 2).

## Discussion

In this study, we have performed an EWAS of lifetime estrogen exposure using the HM450K array and identified 694 CpG sites (FDR *Q* < 0.05) associated with ELEE in the EPIC-Italy study cohort. In addition to this, we have conducted a validation step in a case-control study nested within a large independent cohort, the Generations Study, using targeted bisulfite sequencing. We have developed a methylation index (MI) to predict ELEE using DNA methylation levels at 31 CpG sites and tested the MI for association with breast cancer risk. Although the MI did not correlate with ELEE in the validation cohorts, it was associated with breast cancer risk. Women in the highest quartile of the MI in the Generations Study had 77% higher risk for breast cancer compared with women in the lowest quartile (Additional file 2: Table S5). In the meta-analysis, including three other independent datasets, the highest quartile had a 45% higher risk compared with the lowest quartile (Fig. 4).

There are several potential explanations why the correlation between the MI and ELEE was only seen in the discovery EPIC-Italy dataset and not in the additional validation cohorts. Firstly, it is possible that the observed association between MI and ELEE in EPIC-Italy is a false positive and that the MI was over fitted in this study cohort. It may also be possible that the measured methylation index could capture the biological effects of unknown confounders that are not included in the calculated ELEE model. Alternatively, population-specific differences between the cohorts (or between sub-cohorts of EPIC-Italy) cannot be excluded. For example, there are more smokers in the EPIC studies compared with the Generations Study and MCCS, which considerably affects DNA methylation. Also, there are different patterns in breastfeeding, number of pregnancies, and OC and HRT use across the studies, which may all affect the total lifetime estrogen exposure and DNA methylation. With the current evidence, we conclude that the methylation index developed does not directly predict ELEE.

We observed no evidence for reverse causation with a higher association between the MI and breast cancer risk for cases with a longer time to diagnosis (Fig. 4). In the meta-analysis, the association with breast cancer risk was only significant for the cases with the time to diagnosis greater than the median in the continuous MI model (OR = 1.05, 95% CI 1.01–1.10, *P* = 0.021, Additional file 2: Table S8). More work is needed using longitudinal studies to understand the dynamics of this MI over time.

In this study, we used an ELEE model based on a woman’s age at recruitment (premenopausal women) or age at menopause (postmenopausal women), age at menarche, number of pregnancies, and breastfeeding duration. The model does not include all the variables that can affect estrogen exposure, for example, menstrual cycle regularity, long-term pregnancies with miscarriage or abortion as outcome, and current use of HRT or OC. Hormonal risk factors are difficult to assess comprehensively via questionnaires; for example, the short-term outcome of being pregnant is increased estrogen levels and breast cancer risk, but in the long term, the estrogen levels and risk are reduced compared with nulliparous women [9, 10, 45]. Subtracting 1 year for each pregnancy instead of 9 months for each pregnancy did not materially change the results and might reflect the lifetime estrogen exposure better because there is a delay of 3 months on average before ovulatory cycling resumes. OC and HRT use is associated with elevated risk in current users, but it is not known how it will contribute to the lifetime estrogen exposure or DNA methylation. For this reason, we have not included OC and HRT use in our ELEE model. Other hormonal exposures accompanied by estrogens, such as progesterones, were not considered in this study. We acknowledge that the ELEE model is not a perfect model for cumulative estrogen exposure; however, it was hypothesized that if DNA methylation signature could be identified as an intermediate biological phenotype for the exposure, this might be more accurate measurement than questionnaire-based estimates.

We acknowledge the small sample size of the discovery EWAS in EPIC-Italy (*n* = 216) and potential false positive hits due to artefactual inflation of test statistics. We attempted to reduce the likelihood of false positive associations by correcting for multiple testing and restricting the MI signature to CpGs showing the largest changes in DNA methylation. Replication and validation are important steps to identify valid DNA methylation biomarkers. For validation of the MI and breast cancer risk, we used an independent method, targeted bisulfite sequencing, on a case-control study nested within a large independent study cohort (the Generations Study, *n* = 880) and a meta-analysis across four independent study cohorts (*n* = 2374). Another limitation that we identified was that not all 31 CpG sites are present on the updated Illumina HumanMethylation EPIC (850K) array, which precludes the possibility of including 850K studies in this analysis without changing the model.

Breast cancer risk assessment needs further improvement to be able to identify women at low or high risk of developing breast cancer that would warrant a preventive intervention. It remains to be explored if epigenetic signatures, in combination with other existing risk models, polygenic risk scores and breast density measurements, will improve breast cancer risk prediction and stratification. Furthermore, blood sampling is an accessible and less invasive method that is relatively easy to include into population screening. Targeted prevention approaches, including chemoprevention or lifestyle changes, for high-risk women might reduce the breast cancer incidence rate.

## Conclusion

In this study, a DNA methylation signature in blood associated with breast cancer risk was identified. However, the methylation signature, although developed from ELEE associations in the EPIC-Italy cohort, was not associated with lifetime estrogen exposure in the subsequent cohorts analyzed. Further investigation is required to confirm the interaction between estrogen exposure and DNA methylation in blood, and how epigenetic signatures might improve risk assessment models.

## Additional files

Additional file 1: Supplementary material and methods. (DOCX 65 kb)

Additional file 2: Supplementary **Tables S1** to **S9**. (DOCX 118 kb)

Additional file 3: Supplementary **Figures S1–S4**. (DOCX 2232 kb)

## Abbreviations

BMI: Body mass index
ELEE: Estimated lifetime estrogen exposure
EPIC: The European Prospective Investigation into Cancer and Nutrition
EWAS: Epigenome-wide association study
FDR: False discovery rate
HRT: Hormone replacement therapy
IARC: International Agency for Research on Cancer
MCCS: Melbourne Collaborative Cohort Study
MI: Methylation index
OC: Oral contraceptives
QC: Quality control
