# Supplementary material for: Epigenome-wide association study for lifetime estrogen exposure identifies an epigenetic signature associated with breast cancer risk
Source: Clin Epigenetics. 2019 Apr 30;11:66. doi: 10.1186/s13148-019-0664-7 (PMC6492393; doi:10.1186/s13148-019-0664-7)
Supplement: Supplementary file 1 — Supplementary material and methods. (DOCX 65 kb) [file 13148_2019_664_MOESM1_ESM.docx]

**Supplementary Material & Methods**

A summary of the entire workflow, analyses and datasets used in the study can be found in **Figure S2**.

**Study Cohorts**

***EPIC-Italy study cohort***

The EPIC study was designed to investigate diet and cancer with epidemiological data from over 500,000 participants from 23 centers across 10 European countries (1). At the start of this study EPIC-Italy included epidemiological questionnaire data from 32,059 women recruited between 1992 and 1998 with up to 16.8 years of follow-up time (last updated follow-up date July 2012). For the analysis of the association between the ELEE models and breast cancer risk in this study cases were stratified on age at diagnosis of 50 to enrich for ER-positive disease since ER-status was missing for the majority of the subjects, leaving n=31,864 women for this analysis (**Table S1**, Dataset 1). Cox regression (function ‘coxph’ in R package ‘survival’) adjusted for age was used to explore the association between ELEE models and breast cancer risk in EPIC-Italy. For time variable the time since recruitment to date at diagnosis was used for cases and time since recruitment to last follow up date was used for controls.

HM450K array data was measured in peripheral blood DNA in 324 of these women, including 162 incident breast cancer cases and 162 controls matched on age at recruitment ± 5 years and study center (Dataset 2, **Table 2**). Pre-processing of the HM450K data for EPIC-Italy has been described previously (2). Probes overlapping SNPs or with low detection *P* value in more than 20% of samples and subjects with low detection *P* values in more than 5% of the probes were excluded, resulting in 324 subjects and 448,857 probes. Furthermore, cross-hybridizing probes were excluded, using R function ‘rmSNPandCH’ in package ‘DMRcate’, leaving 404,596 probes for the analysis. In the EWAS of ELEE, cases with age at diagnosis < 50 were excluded leaving n=216 for the EWAS (**Table S2**). For the development of the MI, all cases were included in the analysis (n=237, **Table S2**).

***The Generations Study cohort***

The Generations Study cohort from the Institute of Cancer Research has collected epidemiological data and blood samples from approximately 113,000 women in the UK with the primary aim to study breast cancer aetiology (3). HM450K array data was measured in blood at two time points in 2004 and 2010 in 92 healthy women with no registered cancer at follow up (4). The data from the first time point is used in this study for the development of the MI for predicted ELEE. Duration of breastfeeding was missing for the Generations Study HM450K data subjects and not included in the ELEE-model for these subjects. Subjects with missing information of ELEE were excluded (n=27) leaving 65 subjects for the analysis (Dataset 3, **Table S2**).

**EWAS of ELEE in EPIC-Italy**

Beta regression on beta-values was used to investigate DNA methylation levels at individual CpG sites and their association with ELEE; DNA methylation levels were modeled as a dependent variable in a generalized linear model with beta-distributed responses (R function ‘vglm’ in package ‘VGAM’ (5)). Traditionally a linear regression model on approximately normally distributed log2 transformed beta-values (M-values) has been used in a majority of the published EWAS, however the biological interpretation is lost and it skews the analysis to the upper and lower bounds (6, 7). Beta regression is more suitable for beta-distributed rates bound by 0 and 1 and data showing heteroscedasticity (non-constant variance), all features of HM450K beta-values, and has been proposed in recent studies (8, 9). Inflation of *P* values was analyzed by plotting the observed and the expected *P* values against each other in a quantile-quantile (QQ) plot and the inflation factor lambda was calculated in R package ‘bacon’, which is recommended for EWASs (10). Manifest and annotation data were extracted from the R package ‘IlluminaHumanMethylation450kanno.ilmn12.hg19’

**Targeted bisulfite sequencing using the Fluidigm 48.48 Access Array in the Generations Study**

Targeted bisulfite sequencing was carried out for the 880 samples from the Generations Study cohort. A total of 250ng DNA for each sample was bisulfite converted using the Zymo Research EZ-96 DNA Methylation kit (Cambridge Biosciences, D5004) and bisulfite conversion was verified using Long Interspersed Nuclear Elements-1 (LINE-1) quantitative PCR (qPCR) with primers specifically designed to amplify only bisulfite converted DNA. Target regions were amplified using the Fluidigm 48.48 Access Array (11), which allows amplification of up to 48 target regions in 48 samples in one single run using integrated fluidic circuits (IFCs) and pooling of up to 384 samples before sequencing by the addition of specific barcode sequences. The Access Array (Juno LP 48.48 IFC), reagents for the Access Array and barcode library were ordered from Fluidigm and PCR reagents were ordered from Roche (FastStart High Fidelity PCR System). For pooling of batches a common sequence tag was added at the 5’ end of each primer where to specific barcode sequenced can bind, as provided by Fluidigm. Primers were designed using the recommended settings (product size between 150 to 200 base pairs (bp), annealing temperature 59°C to 61°C) with MethPrimer (v 1.0, http://www.urogene.org/methprimer) and Primer3 (v 4.0, <http://primer3.ut.ee>) and ordered from Sigma-Aldrich. Prior to the targeted sequencing primers specificity was validated by PCR and gel electrophoresis. Selection of CpG sites for sequencing was done in an iterative process; some CpG sites were not included in the target panel due to difficulties with primer design, poor performing primers (no amplified or more than one PCR product) or low sequencing coverage. Each Fluidigm-batch consisted of 44 samples (22 matched case-control pairs) and four samples for quality control including one non-template control (NTC). Amplification of products was confirmed by observing the size-distribution of amplified sample pools (15 samples randomly selected per batch) on the BioAnalyser Agilent 2200 TapeStation using the Agilent High Sensitivity D5000 ScreenTape and reagents (Agilent Technologies). The purified, pooled batches were sequenced on the Illumina MiSeq system (paired-end 150bp). The entire workflow including target amplification using the Fluidigm 48.48 Access Array and sequencing was tested on samples from the laboratory, including cell lines MCF7, MCF10A and MCF12A, and 0% and 100% methylated reference DNAs.

**Pre-processing of targeted sequencing data**

**Alignment and extraction of DNA methylation levels**

FastQ-files for each sample were provided from the sequencing facility. The reads were trimmed for quality, with a Phred score of 20 as threshold, and adapter sequences using Trim galore and Trimmomatic. Trim galore uses cutadapt to trim adapter sequences and remove reads of poor quality and reads of length less than 20bp (12). Trimmomatic uses a sliding window and cut off sequences with a low quality in that window (13). Reads were paired and aligned using Bismark and Bowtie2 with the four bisulfite-converted DNA strands as template (14). DNA methylation levels at each CpG sites were extracted using the bismark methylation extractor tool and bedGraph-files were created using the bismark2bedGraph tool. BedGraph-files with methylation levels and coverage were further analyzed in R version 3.3.2.

**Quality control**

On each batch a control sample (Promega female genomic DNA) was included to estimate batch effects by correlating DNA methylation levels between batches. Two replicated samples (one pair) were included to estimate variance in DNA methylation levels (intra-batch effects) and target CpG sites with a mean difference of 20% or more between duplicated samples were excluded. For each batch and target CpG site the number of pairs with coverage < 30 in at least one of the samples was calculated, and if an average of 30% of the pairs had low coverage the target CpG site was excluded prior to the MI analysis.

**Meta-Analysis Methods**

### Study Cohorts

### *The Generations Study*

Targeted bisulfite sequencing was conducted on 880 samples from the Generations Study, whereof 678 passed quality control. The dataset has been described previously (**Table 2**).

### *EPIC-Italy*

HM450K array data for new breast cancer cases and controls have been generated and 236 (118 case-control pairs, **Table S6**) subjects that had not been used previously in this study were available for the meta-analysis. Case-control pairs were matched on age ± 5 years and study center.

### *EPIC-IARC*

Participants from the EPIC cohort from IARC (International Agency for Research on Cancer) were selected for a nested case-control study. This included subjects from Germany, Greece, Italy, Spain, The Netherlands and UK (15). Subjects overlapping with EPIC-Italy were excluded prior to the analysis. Questionnaire data, anthropometric measurements and blood samples were collected for all study participants at time at recruitment between 1993 and 1998, after ethical review by the IARC. The cancer diagnosis was updated from cancer registries for Italy, Spain, the Netherlands and UK, and by active follow-up for Germany and Greece. The case-control pairs were matched on center, age ± 2 years, time of blood collection, fasting status, menopausal status, current pill and hormone replacement therapy use, and menstrual cycle. A total of 840 (420 case-control pairs, **Table S6**) subjects were included in the meta-analysis. The EPIC-IARC analysis was conducted by Srikant Ambatipudi (IARC).

### *Melbourne Collaborative Cohort Study (MCCS)*

Participants from the MCCS were selected for a nested case-control study. The MCCS is a prospective cohort study including 24,469 women recruited between 1990 and 1994 (16). Cancer incidences were identified via the Victorian cancer registry (VCR) and the Australian Cancer Database, and a total of 680 cases (2.8%) were diagnosed with invasive breast cancer between enrolment and before 2008. In the meta-analysis, 310 cases-control pairs are included, matched on year of birth, year of baseline attendance, and country of origin (**Table S6**). The DNA was extracted from dried blood spots. The MCCS analysis was conducted by Laura Baglietto.

### HM450K array data pre-processing

The following protocol was used for HM450K array data for new EPIC-Italy subjects, EPIC-IARC and MCCS. Pre-processing was conducted in the R package ‘minfi’ (17). Raw intensity data IDAT files were imported into R and background subtraction and normalization were conducted using the ‘preprocessIllumina’ function, followed by correction for type I and II probe bias by subset-quantile within array normalization (SWAN) using the ‘preprocessSWAN’ function (18). Samples with missing information (*P* detection value > 0.01) for more than 5% of the CpG sites were excluded, as well as CpG sites with missing information in 20% or more of the samples. Furthermore, CpG probes located on the Y chromosome and cross-hybridizing probes (19) were excluded using the R package ‘DMRcate’. WBC composition was estimated using the Houseman method (20).

### Statistical analysis

The association between the MI and breast cancer risk were individually analyzed in the four studies using conditional logistic regression (function ‘clogit’ in R package ‘survival’) for breast cancer case-control status and the MI, adjusted by age, BMI, smoking duration, and alcohol consumption (reported at recruitment), and WBC composition. Smoking duration was not adjusted for in EPIC-IARC due to missing data for a majority of the subjects. Alcohol consumption was reported as average units/week for the Generations Study, average self-reported intensity (numbers 1 to 9) at ages 20, 30, 40 and 50 for EPIC-Italy, and average gram per day for EPIC-IARC and MCCS. The association was analyzed for the MI as a continuous variable and also by control-quartiles in each study cohort. Also, the pairs were stratified into two groups on median time to diagnosis to explore differences in the risk association between a shorter or a longer time between blood sampling and diagnosis. The correlation between the MI and ELEE was explored using Pearson’s correlation coefficient.

**References**

1. Riboli E, Hunt KJ, Slimani N, Ferrari P, Norat T, Fahey M, et al. European Prospective Investigation into Cancer and Nutrition (EPIC): study populations and data collection. Public health nutrition. 2002;5(6b):1113-24.

2. van Veldhoven K, Polidoro S, Baglietto L, Severi G, Sacerdote C, Panico S, et al. Epigenome-wide association study reveals decreased average methylation levels years before breast cancer diagnosis. Clinical epigenetics. 2015;7:67.

3. Swerdlow AJ, Jones ME, Schoemaker MJ, Hemming J, Thomas D, Williamson J, et al. The Breakthrough Generations Study: design of a long-term UK cohort study to investigate breast cancer aetiology. Br J Cancer. 2011;105(7):911-7.

4. Flanagan JM, Brook MN, Orr N, Tomczyk K, Coulson P, Fletcher O, et al. Temporal stability and determinants of white blood cell DNA methylation in the breakthrough generations study. Cancer Epidemiol Biomarkers Prev. 2015;24(1):221-9.

5. Ferrari S, Cribari-Neto F. Beta Regression for Modelling Rates and Proportions. Journal of Applied Statistics. 2004;31(7):799-815.

6. Du P, Zhang X, Huang CC, Jafari N, Kibbe WA, Hou L, et al. Comparison of Beta-value and M-value methods for quantifying methylation levels by microarray analysis. BMC bioinformatics. 2010;11:587.

7. Saadati M, Benner A. Statistical challenges of high-dimensional methylation data. Statistics in medicine. 2014;33(30):5347-57.

8. Campanella G, Polidoro S, Di Gaetano C, Fiorito G, Guarrera S, Krogh V, et al. Epigenetic signatures of internal migration in Italy. International journal of epidemiology. 2015;44(4):1442-9.

9. Plusquin M, Guida F, Polidoro S, Vermeulen R, Raaschou-Nielsen O, Campanella G, et al. DNA methylation and exposure to ambient air pollution in two prospective cohorts. Environment international. 2017;108:127-36.

10. van Iterson M, van Zwet EW, Heijmans BT. Controlling bias and inflation in epigenome- and transcriptome-wide association studies using the empirical null distribution. Genome biology. 2017;18(1):19.

11. Adamowicz M, Maratou K, Aitman TJ. Multiplexed DNA Methylation Analysis of Target Regions Using Microfluidics (Fluidigm). Methods in molecular biology (Clifton, NJ). 2018;1708:349-63.

12. Babraham Bioinformatics. Trim Galore! 2018. <https://www.bioinformatics.babraham.ac.uk/projects/trim_galore/>. Accessed: March 2018.

13. Bolger AM, Lohse M, Usadel B. Trimmomatic: a flexible trimmer for Illumina sequence data. Bioinformatics (Oxford, England). 2014;30(15):2114-20.

14. Krueger F, Andrews SR. Bismark: a flexible aligner and methylation caller for Bisulfite-Seq applications. Bioinformatics (Oxford, England). 2011;27(11):1571-2.

15. Ambatipudi S, Horvath S, Perrier F, Cuenin C, Hernandez-Vargas H, Le Calvez-Kelm F, et al. DNA methylome analysis identifies accelerated epigenetic ageing associated with postmenopausal breast cancer susceptibility. European journal of cancer (Oxford, England : 1990). 2017;75:299-307.

16. Severi G, Southey MC, English DR, Jung CH, Lonie A, McLean C, et al. Epigenome-wide methylation in DNA from peripheral blood as a marker of risk for breast cancer. Breast cancer research and treatment. 2014;148(3):665-73.

17. Aryee MJ, Jaffe AE, Corrada-Bravo H, Ladd-Acosta C, Feinberg AP, Hansen KD, et al. Minfi: a flexible and comprehensive Bioconductor package for the analysis of Infinium DNA methylation microarrays. Bioinformatics (Oxford, England). 2014;30(10):1363-9.

18. Maksimovic J, Gordon L, Oshlack A. SWAN: Subset-quantile within array normalization for illumina infinium HumanMethylation450 BeadChips. Genome biology. 2012;13(6):R44.

19. Chen YA, Lemire M, Choufani S, Butcher DT, Grafodatskaya D, Zanke BW, et al. Discovery of cross-reactive probes and polymorphic CpGs in the Illumina Infinium HumanMethylation450 microarray. Epigenetics. 2013;8(2):203-9.

20. Houseman EA, Accomando WP, Koestler DC, Christensen BC, Marsit CJ, Nelson HH, et al. DNA methylation arrays as surrogate measures of cell mixture distribution. BMC bioinformatics. 2012;13:86.
