# Supplementary material for: Epigenome-wide association study for lifetime estrogen exposure identifies an epigenetic signature associated with breast cancer risk
Source: Clin Epigenetics. 2019 Apr 30;11:66. doi: 10.1186/s13148-019-0664-7 (PMC6492393; doi:10.1186/s13148-019-0664-7)
Supplement: Supplementary file 2 — Supplementary Tables S1 to S9. (DOCX 118 kb) [file 13148_2019_664_MOESM2_ESM.docx]

**Supplementary Tables**

**Table S1. Table of characteristics for EPIC-Italy epidemiological data (Dataset 1).**

|  | | **EPIC-Italy** | | |
| --- | --- | --- | --- | --- |
|  |  | **All** | **Cases** | **Controls** |
|  |  | **(n=31,864)** | **(n=1,193)** | **(n=30,671)** |
| **Age** | mean (st.dev.), yrs | 50.7 (8.1) | 53.2 (6.8) | 50.6 (8.1) |
| **Time to diagnosis** | mean (st.dev.), yrs | 8.2 (4.2) | 8.2 (4.2) | NA |
| **Menopausal status** | n (%) |  |  |  |
| *Premenopausal* | | 15563 (48.8%) | 477 (40.0%) | 15086 (49.2%) |
| *Postmenopausal* | | 15866 (49.8%) | 703 (58.9%) | 15163 (49.4%) |
| **Age at menarche** | mean (st.dev.), yrs | 12.5 (1.5) | 12.5 (1.4) | 12.5 (1.5) |
| **Age at menopause** | mean (st.dev.), yrs | 48.3 (4.9) | 48.9 (4.9) | 48.3 (4.9) |
| **Number of pregnancies** | mean (st.dev.) | 1.9 (1.1) | 1.8 (1.0) | 1.9 (1.1) |
| **Ever breastfed** | n (%) | 22773 (71.5%) | 835 (70.0%) | 21938 (71.5%) |
| **Breastfeeding duration** | mean (st.dev.), yrs | 0.7 (0.6) | 0.7 (0.6) | 0.7 (0.6) |
| **BMI** | mean (st.dev.), kg/m^2^ | 25.8 (4.4) | 26.3 (4.5) | 25.8 (4.3) |
| **Alcohol consumption ^a^** | mean (st.dev.), average g/day | 2.6 (2.7) | 2.4 (2.7) | 2.6 (2.7) |
| **Smoking status** | n (%) |  |  |  |
| *Smoker* | | 8240 (25.9%) | 285 (23.9%) | 7955 (25.9%) |
| *Former* | | 6323 (19.8%) | 223 (18.7%) | 6100 (19.9%) |
| *Never* | | 16888 (53.0%) | 672 (56.3%) | 16216 (52.9%) |
| **Smoking duration** | mean (st.dev.), yrs | 6.5 (11.4) | 6.9 (12.2) | 6.5 (11.4) |
| **OC ever** | n (%) | 12871 (40.4%) | 437 (36.6%) | 12434 (40.5%) |
| **OC duration** | mean (st.dev.), yrs | 1.6 (3.1) | 1.5 (3.1) | 1.6 (3.2) |
| **HRT ever** | n (%) | 4961 (15.6%) | 222 (18.6%) | 4739 (15.5%) |
| **HRT duration** | mean (st.dev.), yrs | 0.3 (1.2) | 0.4 (1.2) | 0.3 (1.2) |

^a^ Alcohol consumption intensity was calculated as average reported intensity (1-9) from beer, wine, and spirits at ages 20, 30, 40, and 50.

std.dev = standard deviation

yrs = years

OC = Oral Contraceptives

HRT = Hormone Replacement Therapy

**Table S2. Table of characteristics for HM450K datasets included in the EWAS and meta-analysis.**

|  | | **HM450K data** | | |
| --- | --- | --- | --- | --- |
|  |  | **EPIC-Italy (n=216)** | **EPIC-Italy (n=237)** | **The Generations Study (n=65)** |
|  |  |  |  |  |
| **Incident cases** | n (%) | 97 (44.9%) | 118 (49.8%) | 0 (0%) |
| **Age** | mean (st.dev.), yrs | 54.2 (6.4) | 53.1 (7.2) | 51.8 (8.4) |
| **Time to diagnosis** | mean (st.dev.), yrs | 5.3 (4.5) | 5.4 (4.2) | NA |
| **Menopausal status** | n (%) |  |  |  |
| *Premenopausal* | | 58 (26.9%) | 79 (33.3%) | 32 (49.2%) |
| *Postmenopausal* | | 132 (61.1%) | 132 (55.7%) | 33 (50.8%) |
| **Age at menarche** | mean (st.dev.), yrs | 12.8 (1.5) | 12.7 (1.5) | 12.8 (1.6) |
| **Age at menopause** | mean (st.dev.), yrs | 49.8 (3.6) | 49.7 (3.6) | 48.8 (5.7) |
| **Number of pregnancies** | mean (st.dev.) | 1.7 (1.1) | 1.7 (1.1) | 1.9 (1.1) |
| **Ever breastfed** | n (%) | 180 (83.3%) | 198 (83.5%) | 47 (72.3%) |
| **Breastfeeding duration** | mean (st.dev.), yrs | 0.7 (0.7) | 0.7 (0.6) | NA |
| **BMI** | mean (st.dev.), kg/m^2^ | 25.6 (4.3) | 25.7 (4.3) | 25.4 (3.9) |
| **Alcohol consumption** | mean (st.dev.), average g/day | 6.8 (8.9) | 6.6 (8.7) | 15.6 (12.9) |
| **Smoking status** | n (%) |  |  |  |
| *Smoker* | | 41 (19.0%) | 46 (19.4%) | 2 (3.1%) |
| *Former* | | 42 (19.4%) | 48 (20.3%) | 19 (29.2%) |
| *Never* | | 133 (61.6%) | 143 (60.3%) | 44 (67.7%) |
| **Smoking duration** | mean (st.dev.), yrs | 9.2 (13.3%) | 9.2 (13.1%) | NA |
| **OC ever** | n (%) | 75 (34.7%) | 87 (36.7%) | 50 (76.9%) |
| **OC duration** | mean (st.dev.), yrs | 5.6 (6.0) | 5.0 (5.8) | NA |
| **HRT ever** | n (%) | 35 (16.2%) | 37 (15.6%) | 15 (23.1%) |
| **HRT duration** | mean (st.dev.), yrs | 2.1 (3.0) | 2.0 (2.9) | NA |

std.dev = standard deviation

yrs = years

OC = Oral Contraceptives

HRT = Hormone Replacement Therapy

**Table S3. ELEE models and association with breast cancer risk in EPIC-Italy epidemiological data.**

|  | **All women ^a^** | | **Premenopausal women ^b^** | | **Postmenopausal women ^c^** | |
| --- | --- | --- | --- | --- | --- | --- |
|  | **HR (95% CI)** | ***P* value** | **HR (95% CI)** | ***P* value** | **HR (95% CI)** | ***P* value** |
| Reproductive time | 1.05 (1.04-1.07) | 8x10^-13^ | 1.04 (0.97-1.1) | 0.281 | 1.03 (1.01-1.05) | 4x10^-4^ |
| Reproductive time - number of pregnancies*0.75 | 1.05 (1.04-1.07) | 2x10^-13^ | 1.08 (1.01-1.14) | 0.015 | 1.03 (1.01-1.05) | 4x10^-4^ |
| Reproductive time - number of pregnancies*1 | 1.05 (1.04-1.07) | 1x10-^13^ | 1.08 (1.02-1.14) | 0.007 | 1.03 (1.01-1.05) | 3x10^-4^ |
| Reproductive time - number of pregnancies*1 - breastfeeding duration | 1.05 (1.04-1.07) | 3x10^-12^ | 1.09 (1.03-1.15) | 0.002 | 1.03 (1.01-1.05) | 6x10^-4^ |

^a^ All women: n=30,671 controls and 1,193 cases

^b^ Premenopausal women: n=15,086 controls and 477 cases

^c^ Postmenopausal women: n=15,163 controls and 703 cases

**Table S4. Summary of targeted bisulfite sequencing data in 880 samples in the Generations Study.**

|  | **Sequencing Pool 1**  **(n=288)** | **Sequencing Pool 2**  **(n=240)** | **Sequencing Pool 3**  **(n=240)** | **Sequencing Pool 4**  **(n=192)** | **Sequencing Pool 5**  **(n=96)** |
| --- | --- | --- | --- | --- | --- |
|  | **Batches 1 - 6** | **Batches 7 - 11** | **Batches 12 - 16** | **Batches 17 - 20** | **Batches 12 and 19** |
| Total nr of reads (before pre-processing) | 41,630,730 | 35,243,020 | 37,343,220 | 41,851,010 | 33,058,310 |
| Undetermined reads (reads without barcode sequence) | 733,686  (1.8%) | 818,244  (2.3%) | 838,572  (2.3%) | 894,656  (2.1%) | 646,478  (2.0%) |
| Reads to samples (reads including barcode sequence) | 40,897,040  (98.2%) | 34,424,770  (97.7%) | 36,504,650  (97.8%) | 40,956,350  (98.9%) | 32,411,830  (98.0%) |
| Coverage in targets ^a^ | 1,254 (99.8%) | 1,242 (99.8%) | 1,311 (99.8%) | 1,754 (99.7%) | 3,137 (99.8%) |
| Coverage outside target ^a^ | 2.8 (0.2%) | 2.6 (0.2%) | 2.7 (0.2%) | 5.5 (0.3%) | 5.1 (0.2%) |
| Bisulfite conversion efficacy ^b^ | 99.7% | 99.4% | 99.3% | 99.5% | 99.1% |

^a^ total coverage at each CpG site / observed in number of samples / total nr of CpG site

^b^ bisulfite conversion efficacy calculated as average bisulfite-converted cytosines in a CHG and CHH context (where H = A, T or C).

**Table S5. Quartile analysis of the MI and breast cancer risk**

|  |  | **min** | **max** | **n cases** | **n controls** | **OR (95% CI)** | ***P* value** |
| --- | --- | --- | --- | --- | --- | --- | --- |
| **EPIC-Italy (original)** | Q1 | 27.6 | 31.3 | 41 | 11 | Reference | |
| Dataset 2 | Q2 | 31.3 | 32.5 | 40 | 24 | 1.87 (0.71 – 4.94) | 0.203 |
|  | Q3 | 32.5 | 33.8 | 40 | 46 | 4.14 (1.66 – 10.33) | **0.002** |
|  | Q4 | 33.8 | 39.1 | 41 | 81 | 5.45 (2.17 – 13.67) | **3x10^-4^** |
| **The Generations Study** | Q1 | 20.3 | 30.3 | 66 | 84 | Reference | |
| Dataset 4 | Q2 | 30.3 | 33.8 | 88 | 85 | 1.52 (0.95 - 2.42) | 0.083 |
|  | Q3 | 33.8 | 37 | 82 | 85 | 1.60 (0.96 - 2.64) | 0.069 |
|  | Q4 | 37 | 53.1 | 103 | 85 | 1.77 (1.07 - 2.93) | **0.027** |
| **EPIC-Italy (new) ^a^** | Q1 | 24.7 | 31.9 | 30 | 29 | Reference | |
| Dataset 5 | Q2 | 31.9 | 33 | 30 | 30 | 1.86 (0.57 - 6.07) | 0.303 |
|  | Q3 | 33 | 34.1 | 26 | 29 | 0.83 (0.22 - 3.08) | 0.775 |
|  | Q4 | 34.1 | 43.7 | 32 | 30 | 1.86 (0.54 - 6.38) | 0.325 |
| **EPIC-IARC** | Q1 | 29.4 | 34.9 | 110 | 105 | Reference | |
| Dataset 6 | Q2 | 34.9 | 36.2 | 107 | 105 | 0.95 (0.63 - 1.44) | 0.816 |
|  | Q3 | 36.2 | 37.5 | 93 | 105 | 0.86 (0.56 - 1.31) | 0.476 |
|  | Q4 | 37.5 | 44 | 110 | 105 | 1.07 (0.68 - 1.68) | 0.767 |
| **MCCS** | Q1 | 30.5 | 35.1 | 69 | 78 | Reference | |
| Dataset 7 | Q2 | 35.1 | 36.5 | 63 | 77 | 1.04 (0.63 - 1.69) | 0.891 |
|  | Q3 | 36.5 | 38.2 | 88 | 77 | 1.58 (0.95 - 2.63) | 0.080 |
|  | Q4 | 38.2 | 46.4 | 90 | 78 | 1.69 (0.91 - 3.12) | 0.095 |
| **Meta-analysis ^b^** | Q1 |  |  | 275 | 296 | Reference | |
|  | Q2 |  |  | 288 | 297 | 1.16 (0.88 - 1.52) | 0.290 |
|  | Q3 |  |  | 289 | 296 | 1.22 (0.83 - 1.80) | 0.303 |
|  | Q4 |  |  | 335 | 298 | 1.45 (1.05 - 2.00) | **0.024** |

^a^ EPIC-Italy (new) corresponds to EPIC-Italy samples not included in the development of the MI

^b^ The meta-analysis did not include the original EPIC-Italy data. Results for test of heterogeneity were *Q*=0.40; I^2^=9% for Q2 vs Q1, *Q*=0.17; I^2^=44% forQ3 vs Q1, and *Q*=0.44; I^2^=16% for Q4 vs Q1.

**Table S6. Individual CpG sites and association with breast cancer risk.**

| **HM450K probe** | **Chr** | **Position** | **Nearest gene** | **The Generations Study targeted sequencing data**  **(n=339 pairs)** | | | | | **EPIC-Italy HM450K data**  **(n=162 pairs)** | | | | |
| --- | --- | --- | --- | --- | --- | --- | --- | --- | --- | --- | --- | --- | --- |
|  |  |  |  | **Meth** | **Meth** | **Difference** | **ORperSD (95% CI) ^a^** | **P-value** | **Meth** | **Meth** | **Difference** | **ORperSD (95% CI) ^a^** | **P-value** |
|  |  |  |  | **cases** | **controls** |  |  |  | **cases** | **controls** |  |  |  |
| cg15907392 | chr2 | 80724209 | CTNNA2 | 74.5 | 78.9 | -4.4 | 0.80 (0.67 - 0.95) | 0.012 | 73.7 | 75.6 | -1.9 | 0.68 (0.53 - 0.87) | 0.002 |
| cg10298859 | chr7 | 50849931 | GRB10 | 80.4 | 78.1 | 2.4 | 1.23 (1.02 - 1.49) | 0.033 | 54.5 | 55.4 | -0.9 | 0.62 (0.47 - 0.82) | 0.001 |
| cg12091786 | chr17 | 50465 | RPH3AL | 85.7 | 86.6 | -1.1 | 0.83 (0.69 - 0.99) | 0.036 | 92.4 | 93.5 | -1.1 | 0.70 (0.54 - 0.91) | 0.007 |
| cg26657235 | chr19 | 5568216 | TINCR | 47.6 | 50.1 | -2.4 | 0.77 (0.64 - 0.93) | 0.006 | 47.2 | 48.2 | -1.0 | 0.79 (0.62 - 1.00) | 0.048 |

^a^ The ORs were adjusted for age, BMI, alcohol consumption, and smoking duration (reported at recruitment), and WBC composition.

**Table S7. Table of characteristics for study cohorts in the meta-analysis.**

|  | | **EPIC-Italy (new)** | | | **EPIC-IARC** | | | **MCCS** | | |
| --- | --- | --- | --- | --- | --- | --- | --- | --- | --- | --- |
|  |  | **All (n=236)** | **Cases**  **(n=118)** | **Controls**  **(n=118)** | **All (n=870)** | **Cases**  **(n=435)** | **Controls**  **(n=435)** | **All (n=620)** | **Cases**  **(n=310)** | **Controls**  **(n=310)** |
|  |  |  |  |  |  |  |  |  |  |  |
| **Age** | mean (st.dev.), yrs | 51.2 (7.1) | 51.3 (7.3) | 51.1 (7.0) | 52.2 (9.0) | 52.2 (9.0) | 52.2 (9.0) | 56.7 (8.1) | 56.7 (8.2) | 56.6 (8.1) |
| **Time to diagnosis** | mean (st.dev.), yrs |  | 8.5 (3.7) |  | NA | 7.5 (3.2) | NA | NA | 7.9 (3.8) | NA |
| **Menopausal status** | n (%) |  |  |  |  |  |  |  |  |  |
| *Premenopausal* | | 113 (47.9%) | 61 (51.7%) | 52 (44.1%) | 364 (41.8%) | 182 (41.8%) | 182 (41.8%) | 267 (43.1%) | 131 (42.3%) | 136 (43.9%) |
| *Postmenopausal* | | 123 (52.1%) | 57 (48.3%) | 66 (55.9%) | 506 (58.2%) | 253 (58.2%) | 253 (58.2%) | 353 (56.9%) | 179 (57.7%) | 174 (56.1%) |
| **Age at menarche** | mean (st.dev.), yrs | 12.6 (1.5) | 12.4 (1.4) | 12.8 (1.5) | 13.1 (1.6) | 13.0 (1.7) | 13.2 (1.6) | 13.1 (1.6) | 13.1 (1.6) | 13.1 (1.5) |
| **Age at menopause** | mean (st.dev.), yrs | 47.8 (4.8) | 47.0 (5.0) | 48.5 (4.6) | 48.8 (4.9) | 49.1 (4.7) | 48.5 (5.1) | 49.7 (4.2) | 50.3 (3.8) | 49.2 (4.6) |
| **Number of pregnancies** | mean (st.dev.) | 1.9 (0.9) | 1.9 (1.0) | 1.9 (0.7) | 1.6 (1.1) | 1.5 (1.1) | 1.6 (1.2) | 2.4 (1.7) | 2.5 (1.8) | 2.40 (1.5) |
| **Ever breastfed** | n (%) | 180 (76.3%) | 88 (74.6%) | 92 (78.0%) | 573 (68.2%) | 283 (65.0%) | 290 (66.6%) | 481 (77.6%) | 241 (77.7%) | 240 (77.4%) |
| **Breastfeeding duration** | mean (st.dev.), yrs | 0.6 (0.7) | 0.7 (0.8) | 0.6 (0.6) | 0.6 (0.9) | 0.7 (0.9) | 0.6 (0.8) | 1.0 (1.0) | 1.0 (1.0) | 1.0 (1.0) |
| **BMI** | mean (st.dev.), kg/m^2^ | 26.4 (4.) | 26.3 (4.7) | 26.4 (5.0) | 25.8 (4.5) | 26.0 (4.8) | 25.6 (4.2) | 26.8 (4.7) | 27.2 (5.2) | 26.3 (4.0) |
| **Alcohol consumption^a^** | mean (st.dev.) | 3.0 (1.2) | 3.0 (1.0) | 3.1 (1.3) | 9.1 (12.5) | 9.9 (13.0) | 8.2 (11.9) | 7.4 (12.5) | 6.8 (10.5) | 8.0 (14.3) |
| **Smoking status** | n (%) |  |  |  |  |  |  |  |  |  |
| *Smoker* | | 58 (24.6%) | 27 (22.9%) | 31 (26.3%) | 189 (21.7%) | 90 (20.7%) | 99 (22.8%) | 42 (6.8%) | 21 (6.8%) | 21 (6.8%) |
| *Former* | | 44 (18.6%) | 26 (22.0%) | 18 (15.3%) | 186 (21.4%) | 95 (21.8%) | 91 (20.9%) | 152 (24.5%) | 70 (22.6%) | 82 (26.5%) |
| *Never* | | 134 (56.8%) | 65 (55.1%) | 69 (58.5%) | 491 (56.4%) | 249 (57.2%) | 242 (55.6%) | 426 (68.7%) | 219 (70.6%) | 207 (66.8%) |
| **Smoking duration** | mean (st.dev.), yrs | 10.5 (14.0) | 10.5 (13.8) | 10.5 (14.2) | 9.5 (13.3) | 9.4 (13.2) | 9.6 (13.4) | 8.0 (13.9) | 7.5 (13.5) | 8.5 (14.3) |
| **OC ever** | n (%) | 88 (37.3%) | 43 (36.4%) | 45 (38.1%) | 474 (54.5%) | 237 (54.5%) | 237 (54.5%) | 348 (56.1%) | 163 (52.5%) | 185 (59.7%) |
| **OC duration** | mean (st.dev.), yrs | 4.1 (3.7) | 4.5 (4.1) | 3.6 (3.3) | NA | NA | NA | 6.9 (6.5) | 6.9 (6.6) | 6.9 (6.4) |
| **HRT ever** | n (%) | 46 (19.5%) | 22 (18.6%) | 24 (20.3%) | 223 (25.6%) | 112 (25.7%) | 111 (25.5%) | 189 (30.4%) | 90 (29.0%) | 99 (31.9%) |
| **HRT duration** | mean (st.dev.), yrs | 1.9 (2.2) | 1.8 (2.2) | 2.0 (2.3) | NA | NA | NA | 4.3 (4.8) | 4.4 (4.9) | 4.2 (4.8) |

^a^ alcohol consumption was reported as average units/week for the Generations Study, average self-reported intensity (numbers 1 to 9) at ages 20, 30, 40 and 50 for EPIC-Italy, and average gram per day for EPIC-IARC and MCCS

st.dev = standard deviationyrs = years

**Table S8. Meta-analysis of the MI, breast cancer risk, and time to diagnosis.**

| **Time to diagnosis < median** | **n pairs** | **OR (95% CI) ^b^** | **P*-*value** | ***Q* and I^2^-statistics** |
| --- | --- | --- | --- | --- |
| The Generations Study | 149 | 1.03 (0.97 - 1.09) | 0.315 |  |
| EPIC-Italy ^a^ | 58 | 1.70 (0.77 - 3.72) | 0.188 |  |
| EPIC-IARC | 210 | 0.97 (0.86 - 1.10) | 0.639 |  |
| MCCS | 155 | 1.10 (0.95 - 1.28) | 0.198 |  |
|  |  |  |  |  |
| Meta-analysis | 572 | 1.03 (0.98 - 1.08) | 0.241 | Q=0.36; I^2^=13% |
| **Time to diagnosis > median** | **n pairs** | **OR (95% CI) ^b^** | **P-value** | ***Q* and I^2^-statistics** |
| The Generations Study | 190 | 1.06 (1.01 - 1.12) | **0.024** |  |
| EPIC-Italy ^a^ | 59 | 1.57 (0.85 - 2.91) | 0.148 |  |
| EPIC-IARC | 210 | 1.02 (0.91 - 1.15) | 0.689 |  |
| MCCS | 155 | 1.02 (0.90 - 1.16) | 0.723 |  |
|  |  |  |  |  |
| Meta-analysis | 614 | 1.05 (1.01 - 1.10) | **0.021** | Q=0.55; I^2^=0% |

^a^ EPIC-Italy corresponds to the new EPIC-Italy samples, not included in the development of the MI

^b^ ORs were adjusted for age, BMI, alcohol consumption, and smoking duration at recruitment, and WBC composition.

**Table S9 Correlation between the MI and ELEE.**

|  | **n subjects** | **Correlation coefficient** | ***P* value** |
| --- | --- | --- | --- |
| **The Generations Study** | 678 | -0.04 | 0.340 |
| **EPIC-Italy ^a^** | 233 | 0.07 | 0.263 |
| **EPIC-IARC** | 510 | 0.06 | 0.154 |
| **MCCS** | 436 | -0.04 | 0.449 |

^a^ EPIC-Italy corresponds to the new EPIC-Italy samples, not included in the development of the MI
