# Supplementary material for: Epigenome-wide association study for lifetime estrogen exposure identifies an epigenetic signature associated with breast cancer risk
Source: Clin Epigenetics. 2019 Apr 30;11:66. doi: 10.1186/s13148-019-0664-7 (PMC6492393; doi:10.1186/s13148-019-0664-7)
Supplement: Supplementary file 3 — Supplementary Figures S1–S4. (DOCX 2232 kb) [file 13148_2019_664_MOESM3_ESM.docx]

**Supplementary Figures**

**Figure S1. Probes for estimation of WBC composition.**

**A.** Five probes from the HM450K array included in the Houseman method were identified in EPIC-Italy Dataset 2 (first row) to independently correlate with different WBCs (including B-cells, CD4+T-cells, CD8+T-cells, Monocytes and NK-cells). Similar correlations were seen in the Generations Study HM450K data (Dataset 3, plots in second row). The CpG sites were included in the target panel to estimate WBC composition in the targeted bisulfite sequencing data. **B.** DNA methylation levels at the five CpG sites for WBC estimation in targeted sequencing data. No significant (P > 0.05) differences in DNA methylation levels between cases and controls were observed in the Generations Study (n=328 matched case-control pairs with coverage > 30 for all five CpG sites)

**Figure S2. Datasets and workflow for the study.**

**Figure S3.** **Quantile-quantile (QQ) plot.**

The expected and the observed P values from the EWAS of ELEE in EPIC-Italy (Dataset 2, n=216). Inflation factor lambda was calculated using the R package ‘bacon’.

**Figure S4. Sensitivity analyses for the EWAS of ELEE.**

The main EWAS included n=216 women from EPIC-Italy HM450K data (Dataset 2) where cases with an age at diagnosis < 50 were excluded. To explore the effect of excluding younger cases the same EWAS was run on the 694 significant probes including all cases and controls (**A**, n=237) or controls only (**B**, n=119). The plot shows the comparison of the estimates in the main EWAS (n=216) and the two other subsets of EPIC-Italy. All probes remained significant in the first analysis (n=237) after correction for multiple testing using FDR (Q < 0.05), and 563 in the second analysis (n=119). Blue dots indicate CpG sites included in the targeted sequencing.
